# P05.01. Operationalization and assessment of mindfulness: the perspective of Buddhist clergy and laypersons

**DOI:** 10.1186/1472-6882-12-S1-P361

**Published:** 2012-06-12

**Authors:** M Christopher, V Christopher, S Charoensuk

**Affiliations:** 1 Pacific University, Hillsboro, USA; 2 Portland State University, Portland, USA; 3 Boromarajonani College of Nursing, Chon Buri, Thailand

## Purpose

Preliminary evidence attests to the effectiveness of mindfulness-based interventions in reducing symptoms associated with a variety of medical and psychological conditions. However, there are discrepancies in how mindfulness has been operationalized, assessed, and practiced. Contemporary mindfulness assessments were developed by Western scientists and have questionable validity, particularly among diverse cultural groups. Therefore, in this research we examined mindfulness from the perspective of experts – Theravāda, Tibetan, and Zen Buddhist clergy and lay practitioners.

## Methods

A sequential exploratory mixed methods design was used, the first phase of which involved conducting open-ended interviews with Buddhist clergy and lay practitioners. These qualitative results are reported here (the quantitative phase will begin in summer 2012). We developed a coding schema through a series of steps to identify core categories endorsed across groups. Participants were 36 Buddhist clergy and laypersons (14 Zen, 21 Theravāda, and 1 Tibetan) who completed a qualitative interview and several existing mindfulness measures (we are currently interviewing more Tibetan Monks).

## Results

Although data analysis is ongoing, we identified several categories that were most salient among our participants. These are: returning to the present moment, nonjudgmental awareness, sensory awareness, and impermanence. Although these categories overlap with Western conceptualizations of mindfulness, several areas of divergence also emerged, including, an emphasis on other elements of the Noble Eightfold Path and the distinction between “basic” and “advanced” mindfulness.

## Conclusion

Valid assessment of mindfulness in Western psychology is essential to enhancing our understanding of the many benefits associated with this Buddhist-derived practice and its associated phenomena. Although there are important areas of convergence between contemporary Western and traditional Buddhist conceptualizations of mindfulness, questions regarding the validity of these measures remain. Our progress toward developing a measure of mindfulness in which we integrate elements of existing measures and information gleaned from these interviews will also be discussed.

